# *Toxoplasma gondii* Infection Inhibits Histone Crotonylation to Regulate Immune Response of Porcine Alveolar Macrophages

**DOI:** 10.3389/fimmu.2021.696061

**Published:** 2021-07-08

**Authors:** Jing Yang, Zhengming He, Chengjie Chen, Senyang Li, Jiahui Qian, Junlong Zhao, Rui Fang

**Affiliations:** State Key Laboratory of Agricultural Microbiology, College of Veterinary Medicine, Huazhong Agricultural University, Wuhan, China

**Keywords:** *Toxoplasma gondii*, porcine alveolar macrophage, posttranslation modification, histone crotonylation, immune response

## Abstract

*Toxoplasma gondii* (*T. gondii*) is an obligate intracellular parasite that can infect almost all warm-blooded animals, causing serious public health problems. Lysine crotonylation (Kcr) is a newly discovered posttranslational modification (PTM), which is first identified on histones and has been proved relevant to procreation regulation, transcription activation, and cell signaling pathway. However, the biological functions of histone crotonylation have not yet been reported in macrophages infected with *T. gondii*. As a result, a total of 1,286 Kcr sites distributed in 414 proteins were identified and quantified, demonstrating the existence of crotonylation in porcine alveolar macrophages. According to our results, identified histones were overall downregulated. HDAC2, a histone decrotonylase, was found to be significantly increased, which might be the executor of histone Kcr after parasite infection. In addition, *T. gondii* infection inhibited the crotonylation of H2B on K12, contributing on the suppression of epigenetic regulation and NF-*κ*B activation. Nevertheless, the reduction of histone crotonylation induced by parasite infection could promote macrophage proliferation *via* activating PI3K/Akt signaling pathway. The present findings point to a comprehensive understanding of the biological functions of histone crotonylation in porcine alveolar macrophages, thereby providing a certain research basis for the mechanism research on the immune response of host cells against *T. gondii* infection.

## Introduction

As a member of the phylum Apicomplexa, *Toxoplasma gondii* (*T. gondii*) is an important food-borne and water-borne zoonotic pathogen (1). This obligate intracellular parasite can infect all warm-blooded animals including approximately one third of population around the world (2). Rapidly proliferated tachyzoites stages are usually controlled by immune responses of hosts, thus immunocompetent hosts generally maintain asymptomatic status as bradyzoite cysts after infection (3). However, individuals with immunocompromise or congenital infection may lead to life-threatening disseminated infection such as ophthalmitis, encephalitis, abortion, and even death (4, 5). Current studies have indicated that consumption of undercooked meat infected with *T. gondii* may be one of the primary sources of human toxoplasmosis cases (6, 7), which is considered to account for at least 30% in the route of human toxoplasmosis transmission (8). Pigs are one of the most susceptible hosts of *T. gondii*, and porcine model is proved to be superior to the murine model (9). In addition, the threat of porcine toxoplasmosis to humans is also exposed to great challenges with the growing risk of porcine organ transplantation (10). Considering that *T. gondii* has a serious impact on public health with the development of the pig industry, the prevention and control of swine infected with *T. gondii* are of remarkable significance for the prevention of human toxoplasmosis. Macrophages, essential effector cells, are directly involved in host defense against protozoon infection, which can produce high levels of IL-12 and IFN-γ to inhibit parasite growth in the innate immune response (11, 12). Tachyzoites fail to acidify and rapidly proliferate in normal macrophages, which ultimately leads to the lysis of the host cell (13, 14). In turn, the signaling pathway in infected cells can be interfered by *T. gondii*, allowing the parasite to evade the innate immune response (15). However, research on the mechanism of porcine macrophages in response to *Toxoplasma* infection is still limited.

Protein posttranslational modification (PTM), a crucial protein function regulation, is essential to the structure and function of proteins under physiological and pathological conditions, such as gene expression and regulation, cell growth, embryonic development, metabolism, and disease treatment (16, 17). Lysine crotonylation (Kcr) of both histone and non-histone proteins is a newly identified modification, which exists in mammals and is relevant to active transcription and cell signaling pathway (16, 18). Recent studies have demonstrated that YEATS2, a protein containing the YEATS domain, is a histone crotonylation reader that can directly concatenate histone crotonylation to activate transcription (19). In fact, histone crotonylation is an evolutionarily conserved modification, which presents in a wide range of eukaryotic organisms (20). Nevertheless, the functional impact of histone Kcr in macrophages remains unclear. The regulatory enzymes of histone crotonylation have also been widely investigated. It has been actively studied that classic histone acetyltransferases CBP/p300 and MOF are also involved in the catalytic histone crotonylation (21, 22), and class I histone deacetylases (HDACs) have been found to be the major histone decrotonylases under different conditions (23–25). An increasing body of evidence reveals that HDACs are involved in the modulation of various cell functions, including regulation of inflammatory gene expression, DNA repair, and cell proliferation by altering the status of protein acylation (26, 27). Additionally, previous research reported that the inhibition of class I HDAC1/2/3 leads to histone Kcr significant induction and affects gene expression and epigenome, thereby affecting cell function and regulating immune responses (28). However, it has never been clearly identified whether and how HDACs regulate histone Kcr in macrophages infected with *T. gondii*.

In the present study, a method of LC-MS/MS coupled with highly sensitive affinity enrichment was first applied to quantitatively analyze the differentially expressed Kcr proteins in porcine alveolar macrophages infected with *T. gondii*. Furthermore, *T. gondii* infection induces the overall downregulation of histone crotonylation mediated by HDAC2 in 3D4/21 cells, which is reflected in the inhibition of H2BK12cr epigenetics, thereby suppressing the activation of NF-κB. Unexpectedly, the decrease of histone Kcr modification promotes macrophage proliferation. Our work provides comprehensive insights into the biological functions of histone crotonylation in porcine alveolar macrophages, which may pave the way for exploring the specific mechanisms of histone crotonylation in the immune response of host cells against *T. gondii* infection.

## Experimental Procedures

### Parasites Infection and Sample Collection

Porcine alveolar macrophages (3D4/21 cells) and PK-15 cells were infected with tachyzoites of *T. gondii* type I strain RHΔ*hxgprt* (MOI = 5) propagated in human foreskin fibroblast cells (obtained from ATCC, Maryland, USA) in DMEM supplemented with 2% FBS at 37°C with 5% CO_2_ or mock infected with an equal amount of phosphate-buffered saline (PBS, pH 7.4) (herein referred to as 3D4/21 + RH and Control, PK-15 + RH and Control), respectively. After 24 h post-infection, all samples were harvested and stored at −80°C for subsequent protein and chromatin extraction.

### Western Blot Analysis

The total protein was extracted according to the manufacturer’s protocols. Equal amounts of proteins (20 μg) were separated by 12% SDS-PAGE prior to transferring to PVDF membranes (Millipore, Massachusetts, USA), and then the PVDF membranes were blocked in 2% BSA at room temperature for 2 h. The membranes were incubated with primary antibodies against pan Kcr (1:1,000 dilutions; PTM Bio, PTM-502, Hangzhou, China), H2BK12cr (1:1,000 dilutions; PTM Bio, PTM-509, Hangzhou, China), H2B (1:1,000 dilutions, PTM Bio, PTM-1006, Hangzhou, China), H3 (1:1,000 dilutions, Beyotime, AF7101, Shanghai, China), HDAC1 (1:1,000 dilutions; CST, 5356, MA, USA), HDAC2 (1:1,000 dilutions; CST, 5113, MA, USA), Akt (1:1,000 dilutions, CST, 4691, MA, USA), p-Akt (Ser473) (1:1,000 dilutions, CST, 4060, MA, USA), NF-*κ*B-p65 (1:1,000 dilutions, CST, 6956, MA, USA), *β*-actin (1:1,000 dilutions, CST, 4970, MA, USA), HDAC3 (1:1,000 dilutions, Abcam, ab187139, Cambridge, UK) and HDAC8 (1:1,000 dilutions, Abcam, ab32369, Cambridge, UK) at 4°C overnight. Subsequently, the membranes were detected with secondary antibodies (1:2,000 dilutions; Beyotime, Shanghai, China) at room temperature for 1 h after washing with TBST five times. Eventually, the protein signal was detected with an ECL Plus Western Blotting Detection System (Image Quant LAS 4000mini, United States).

### TMT Labeling and Quantitative Proteomics Analysis

The TMT labeling crotonylation quantitative proteomics analysis was performed by Jingjie PTM BioLab Co. Ltd (China). The digested peptide was desalted by Strata X C18 (Phenomenex, CA, USA) and vacuum freeze-dried, and then the peptide was labeled according to manufacturer’s protocol for TMT kit. Briefly, the labeling reagent was dissolved in acetonitrile. The mixtures were incubated for 2 h at room temperature, desalted, and dried by vacuum centrifugation. In order to enrich crotonylated modified peptides, the peptides were dissolved in IP buffer (100 mM NaCl, 1 mM EDTA, 50 mM Tris-HCl, 0.5% NP-40, pH 8.0), and the supernatant was incubated with pre-washed crotonylated resin beads (PTM Bio, Hangzhou, China) at 4°C overnight with gentle shaking. After incubation, the resin beads were washed with IP buffer four times and deionized water twice. Eventually, the beads-bound peptides were eluted with 0.1% trifluoroacetic acid, and the collected and vacuum dried eluate was desalted with C18 ZipTips (Millipore) according to the manufacturer’s instructions for subsequent LC-MS/MS analysis.

LC-MS/MS was performed as described previously (20, 29). Briefly, peptides were dissolved in solvent A (0.1% formic acid), and peptide separation was conducted using an EASY-nLC 1000 ultra-performance liquid chromatography system. The liquid phase gradient was as follows: 7 to 22% solvent B (0.1% formic acid in 98% acetonitrile) for 26 min, 22 to 35% for 8 min and increased from 35 to 80% over 8 min then hold at 80% at the last 3 min; all flow rates were maintained at 350 nl/min. Subsequently, the separated peptides were ionized using NSI source (voltage was 2.0 kV) and then analyzed by Orbitrap Fusion™ mass spectrometry. A data-dependent scanning (DDA) program was applied to acquire the data. To improve the efficient utilization of mass spectrometry, the automatic gain control (AGC) was set at 5E4 and the signal threshold is set to 5,000 ions/s.

A Maxquant search engine (v.1.5.2.8) was employed to search secondary mass spectrometry data against UniProt *Sus scrofa* database (40,708 sequences) concatenated with reverse decoy database, which was added to calculate the false positive rate (FDR). The cleavage enzyme was set to Trypsin/P allowing a maximum of four missing cleavages, a minimum peptide length of seven amino acid residues, and a maximum of five modified peptides. The mass error tolerance for precursor ions was set as 20 ppm in First search and 5 ppm in Main search, respectively, and the mass error tolerance for secondary fragment ions was set as 0.02 Da. Furthermore, carbamidomethyl on cysteine was specified as fixed modification; oxidation on methionine, deacylation on asparagine and glutamine, acetylation on protein N-terminal, and crotonylation on lysine were specified as variable modifications. TMT-6plex was the method for quantification, FDR values set less than 1% were used for identification of both modified proteins and peptides, and minimum score for modified peptides was set >40.

### Bioinformatics Analysis

Soft MoMo (http://meme-suite.org/tools/momo) was performed to analyze the model of peptide sequences that comprised amino acids at specific positions of modified 21-mers (10 amino acids upstream and downstream of the site) for all proteins. Minimum number of occurrences was set to 20 with *p* value <0.000001. Secondary structures were predicted using NetSurfP. Subcellular localization was predicted by Wolfpsort. Eukaryotic Orthologous Group (KOG) annotation was obtained from the NCBI-COG database (https://www.ncbi.nlm.nih.gov/COG/). GO term and protein domain enrichment were performed by a two-tailed Fisher’s exact test that was used to test the enrichment of the differentially modified protein against all identified proteins, and a corrected *p*-value <0.05 was considered significant. Differentially modified protein database accession or sequences were searched against the STRING (v.10.5) protein network interaction database. The differential modified protein interaction was obtained according to a confidence score >0.7 (high confidence). Interaction network form STRING was visualized in R package “networkD3”.

### Histone Extraction

Histone-enriched fractions were extracted from 3D4/21 cells using an acid extraction method (23, 30). Briefly, the cell pellet was resuspended with lysis buffer (10 mM Tris–HCl pH 8.0, 1 mM KCl, 1.5 mM MgCl_2_, 1 mM DTT, 2 mM PMSF, and protease inhibitors) and then incubated at 4°C for 1 h. Subsequently, the nucleus was pelleted by centrifuging at 10,000×g for 10 min at 4°C. Histones were harvested by adding 0.4 N H_2_SO_4_ to resuspend nucleus, and 100% trichloroacetic acid (trichloroacetic acid final concentration 33%) was added to precipitate histones. Finally, the precipitated histones were pelleted at 16,000×g for 10 min at 4°C and washed with ice cold acetone twice. The collected samples were stored at −80°C for subsequent analysis.

### ChIP and ChIP-Seq Assays

The chromatin immunoprecipitation and ChIP-seq were performed by Wuhan igenebook Biotechnology Co. Ltd (China). Briefly, 3D4/21 cells were cross-linked with 1% formaldehyde for ran chromatin extraction. After sonication, fragmented DNA was obtained and then incubated with antibody (anti-H2BK12cr)-coated beads for 12 h at 4°C. After extensive washing, 20 μl 5M Nacl was added into immunoprecipitated chromatin to de-cross-linked overnight, and the concentration of purified de-cross-linked product was measured for subsequent sequencing. Enriched DNA fragments were subjected to library preparation and sequencing was constructed on Illumina HiSeq 2000 with PE 150 method. Clean data were filtrated using FastQC (version: 0.11.5) and mapped to the reference genome (https://www.ncbi.nlm.nih.gov/genome/84) by BWA (version: 0.7.15-r1140). ChIPseeker R package was applied to draw vennpie diagram of the distribution of Reads on gene functional elements. Deeptools (version: 2.5.4) was used to describe the read density distribution. MACS was used to analyze peak information in the genome, and the threshold for screening the significant Peak was a value of q <0.05. Peaks with FDR value <0.05 and Fold > or <0 in both replications were considered as differentially expressed peaks, and Integrated Genome Viewer was utilized to generate signal plotting of individual genes (31).

### Quantitative Real-Time Polymerase Chain Reaction Assay

The total RNA was extracted using Trizol reagent (Transgen Biotech, Beijing, China) following the manufacturer’s protocols. Subsequently, the total RNA (1 mg) was reverse-transcribed to cDNA using a reverse transcription kit (Vazyme, Nanjing, China). Gene-specific primers for quantitative real-time PCR were designed using Premier 7.0 software (Premier Biosoft International, Palo Alto, CA, United States); the gene-specific quantitative real-time PCR primes are listed in **Table 1**. Quantitative RT-PCR was performed on ABI Step one plus real-time PCR instrument using SYBR Green qPCR Master Mix (Vazyme, Nanjing, China). The expression levels of target genes were normalized to *β*-tubulin levels using the 2^-ΔΔCt^ method.

**Table 1:** Primers used for qRT-PCR.

Name	Primer sequence (5′–3′)	GenBank accession number	Product size (bp)
HDAC1	CGCATGACTCACAATTTGCT AGCCATCAAATACCGGACAG	XM_013999116	211
HDAC2	ACAGGAGACTTGAGGGAT CACATTTAGCGTGACCTT	XM_001925318	232
HDAC3	GCTGCTGGACGGATGAGA CTGGATGGAGCGTGAAGT	NM_001243827	108
HDAC8	Ccattaaagtatctcaaggc gtgggccaatgtgattggtg	XM_021080458.1	227
*β*-actin	TCCACGAAACTACCTTCAACTCCAA GATCTCCTTCTGCATCCTGTC	XM_003124280.5	131

### Cell Transfection and Nucleus–Cytoplasmic Fractionation

Porcine alveolar macrophages were transfected with small interfering RNA or corresponding SiRNA NC (GenePharma, Shanghai, China) using Exfect Transfection Reagent (Vazyme, Nanjing, China) according to the manufacturer’s instructions. The cells were harvested 36 h after transfection for subsequent experiments. Nuclear–cytoplasmic fractionation was conducted using the NE-PER Nuclear and Cytoplasmic Extraction Reagents kit (Thermo Fisher Scientific, 78833, MA, USA) according to the manufacturer’s protocol.

### Cell Proliferation Assays

Cell proliferation was determined using CCK-8 (Vazyme, Nanjing, China) and EdU incorporation assays (Beyotime, Shanghai, China), respectively. For CCK-8 assay, 10 μl CCK-8 solution was added to each well after treatment. After 1 h of continuous incubation, the optical density (OD) of each well was measured at wavelength of 450 nm on a microplate reader (Bio-Tek, Vermont, USA). EdU incorporation assay was conducted according to the manufacturer’s protocol. Briefly, treated cells were incubated with EdU for 2 h and then subjected to EdU immunostaining. EdU positive cells were determined using a fluorescence microscope (BX53, Olympus, Japan) and CytoFLEX LX flow cytometer (Beckman Coulter, Brea, CA, USA), respectively.

### Statistical Analysis

All values were performed with GraphPad Prism 6 (GraphPad Software Inc., La Jolla, CA, USA) and presented as the mean ± SEM. The statistical differences between groups were determined using *Student’s t*-test. A value of *p <*0.05 was defined as statistically significant.

## Results

### Global Analysis of Kcr Proteome in Porcine Alveolar Macrophages

**Figure 1:**
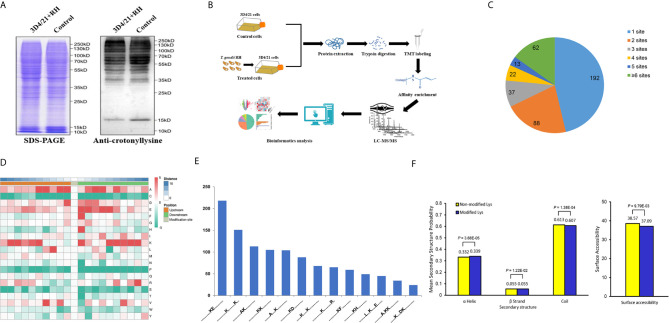
Global analysis of Kcr proteome in porcine alveolar macrophages. **(A)** SDS-PAGE and Western blot analysis of the total protein content of porcine alveolar macrophages infected with *T. gondii* and the control group probed with anti-crotonyllysine. **(B)** Schematic representation of experimental workflow for TMT quantification of Kcr in 3D4/21+RH and control cells. **(C)** Pie chart revealing the distribution of the number of identified Kcr sites per protein. **(D)** Heat map showing the frequency of the different of amino acids around the Kcr. **(E)** Number of the crotonylated peptides in each motif. **(F)** Distribution of lysine crotonylation in different protein secondary structures and the predicted surface accessibility of Kcr sites.

The existence and difference of protein modification in porcine alveolar macrophages were determined by Western blot with anti-crotonyllysine antibody (**Figure 1A**). Additionally, a proteomic method based on immunoaffinity enrichment and high-resolution LC-MS/MS with two replications was applied to quantify differentially expressed crotonylated proteins and modification sites (**Figure 1B**). In the present study, 56,763 secondary spectra were obtained, and the number of available and effective spectrum was 3,871 (6.82%) after searching the library of protein theoretical data by mass spectrometry secondary spectrum; in addition, 1,896 peptides and 1,542 crotonylated peptides were identified (**Figure S1A**). In order to further verify the MS data, we first conducted the detection of the mass error of these identified peptides, and the distribution of peptide mass error was near zero, which proved the accuracy of the MS data (**Figure S1B**). The length of most peptides ranged from 7 to 22 amino acid residues in line with the attribute of tryptic peptides, indicating the high quality of protein samples (**Figure S1C**). Then on the basis, a total of 1,557 unique crotonylation sites in 458 proteins were identified, and among which 1,286 Kcr sites in 414 proteins were quantified. Among these Kcr proteins, 192 (46.4%) had a single Kcr site, which accounts for the majority, and 62 (15.0%) had more than six Kcr sites (**Figure 1C** and **Table S1**).

To explore the sequence properties of Kcr sites and presume the specific motifs, we performed the MoMo software to analyze the enriched motifs in all identified crotonylated proteins. LXXKcrXXXXE, KcrE, AXKcrKcr, KcrXXXDKcr, KcrXXXXXXXKcr, AKcr, AXXXKcr, KcrF, KcrD, KcrH, KcrKcr, KcrXXXXXXXXR, and KXXXXKcr were identified with different abundances (**Figures 1D, E** **)**, and these motifs were distinct from the reported motifs of Kcr in other PTMs (32, 33). We next applied NetSurfP to investigate the relationship between lysine crotonylation and protein secondary structures; the structural analysis revealed that approximately 33.9% of Kcr sites were identified in *α*-helices, 5.5% of Kcr sites were found in *β*-strand, and the rest of 60.7% were in disordered coils (**Figure 1F**). Consideration of no remarkable difference of distribution pattern between all protein lysine residues and crotonylated lysine residues implies that there was no preference on structure for Kcr in 3D4/21 cells after *T. gondii* infection. Similarly, the surface accessibility of Kcr showed no significant difference from the total protein lysine residues (**Figure 1F**), suggesting the surface attributes of modified protein residues may not be affected by Kcr.

### Quantitative Analysis of Lysine Crotonylation in Porcine Alveolar Macrophages After *T. gondii* Infection

**Figure 2:**
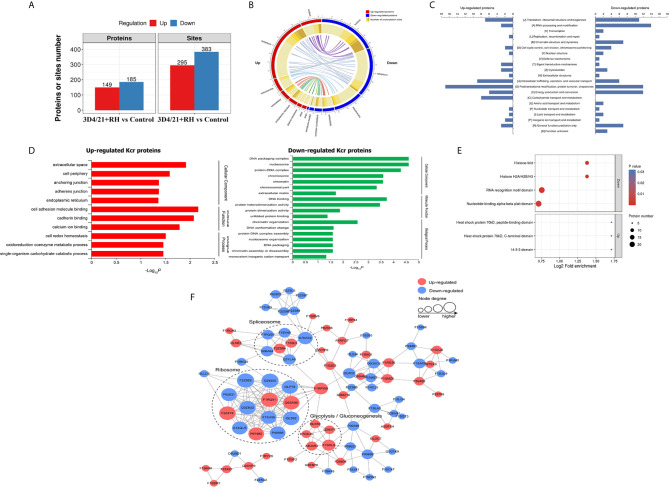
Quantitative analysis of lysine crotonylation in porcine alveolar macrophages after *T. gondii* infection. **(A)** The differentially expressed Kcr proteins between 3D4/21+RH and control cells. **(B)** The differentially crotonylated proteins were classified based on subcellular location between 3D4/21+RH and control cells. **(C)** The differentially crotonylated proteins were analyzed by the KOG (Eukaryotic Orthologous Groups) database. **(D)** Bar graphs showing GO-based enrichment analysis of the differentially crotonylated proteins between 3D4/21+RH and control cells. **(E)** Bubble diagram showing protein domain enrichment analysis of the differentially crotonylated proteins between 3D4/21+RH and control cells. **(F)** Interaction networks of all identified Kcr proteins based on the STRING database.

We next quantitatively analyzed the changes of protein Kcr in response to *T. gondii* infection in comparison with total protein abundances in 3D4/21 cells. Modification sites in both two replicates with *p <*0.05 were considered as significant different modification, and the standard for up- or downregulated modification sites was set as a fold change >1.2 or <0.83, respectively. Based on the above data and standards, 295 modification sites in 149 proteins were upregulated, and 383 modification sites in 185 proteins were downregulated in 3D4/21+RH *vs*. control group (**Figure 2A** and **Table S2**).

The subcellular location analysis of quantifiable proteins manifested that most of upregulated modified proteins were predicted to be in the cytoplasm (52%) and nucleus (16%), and 11% of identified proteins were related to mitochondria. In terms of downregulated proteins, three main types of subcellular components were also associated with the nucleus (35%), cytoplasm (32%), and mitochondria (18%) (**Figure 2B**). Overall KOG analysis of differentially modified proteins in RH+3D4/21 *vs*. control group was conducted using NCBI database, and the results revealed that the largest group of differentially modified proteins were functionally clustered in the categories of posttranslational modification, protein turnover, chaperones, translation, ribosomal structure and biogenesis, and RNA processing and modification (**Figure 2C**).

In order to better clarify the potential function of lysine crotonylation and the biological function of differentially modified proteins in 3D4/21 cells after *T. gondii* infection, we next performed GO enrichment analysis of differentially modified proteins on three major categories (biological processes, cellular components, and molecular function). In terms of downregulated crotonylated proteins, the significant biological processes were chromatin organization, DNA conformation change, and protein–DNA complex assembly, while a large proportion of upregulated modified proteins were involved in cell redox homeostasis, oxidoreduction coenzyme metabolic process, and single-organism carbohydrate catabolic process (**Figure 2D**). The above results indicated that Kcr proteins played essential functions in diverse biological processes in macrophages after *T. gondii* infection. Most of the enriched proteins in protein domain enrichment analysis were downregulated, which were involved in nucleotide-binding alpha-beta plait domain, RNA recognition motif domain, histone H2A/H2B/H3, and histone-fold (**Figure 2E**). The results of protein–protein interaction (PPI) analysis revealed that Kcr levels on most ribosome and spliceosome-related proteins were downregulated, which indicated that *T. gondii* infection might negatively regulate Kcr in macrophages (**Figure 2F**).

### *T. gondii* Infection Negatively Regulates Histone Kcr in Porcine Alveolar Macrophages

**Figure 3:**
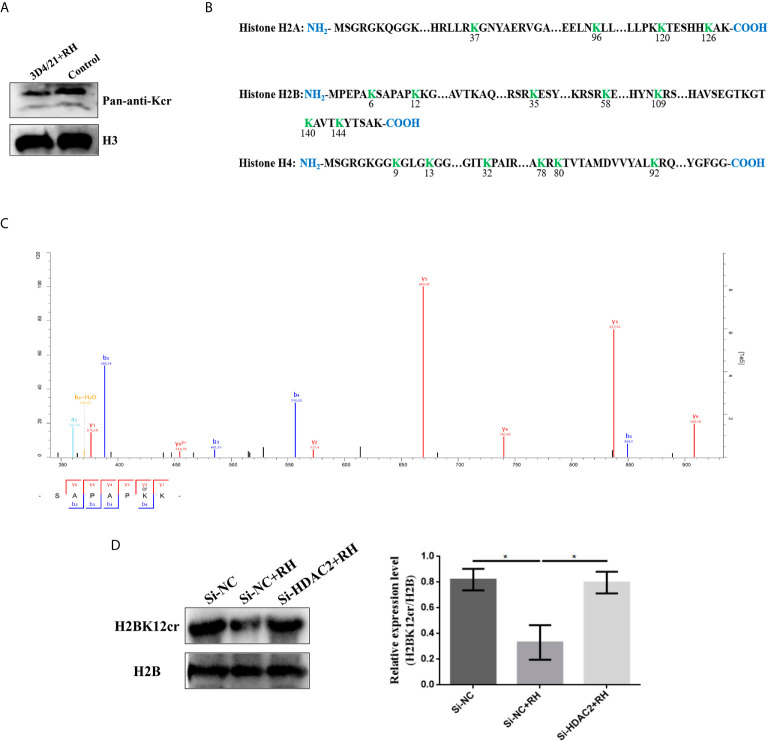
*T. gondii* infection negatively regulates histone crotonylation in porcine alveolar macrophages. **(A)** Detection by immunoblotting of Kcr in histone isolated from control and 3D4/21+RH cells. **(B)** Histone Kcr sites detected by quantitative proteomics analysis. **(C)** Full MS and MS/MS spectra for K12cr of H2B. **(D)** Western blot analysis of the expression levels of H2BK12cr. The data are presented as the mean ± SEM of three independent experiments. *p < 0.05, one-way ANOVA.

Histone PTMs have been proved to play a vital role in numerous biological processes, such as cell growth, differentiation, and epigenesis (34, 35). Lysine is an evolutionarily conserved histone posttranslational modification marker, which has been suggested as a robust indicator of active promoters in histones (16). Hence, we focused on lysine crotonylation of histones, and it could be observed from a map of canonical histones with crotonylation that a total of 17 histone Kcr sites were identified and quantified. These identified histones were mainly distributed in histone H2A, histone H2B, histone H4, and all identified sites presented significant decreases in crotonylation levels, which was consistent with the changes of Kcr levels of histones in the Western blot results (**Figures 3A, B** **)**. Next, we thus focus on H2BK12cr to investigate the regulatory effect of histone Kcr in macrophages infected with *T. gondii*. LC-MS/MS detected a 0.67-fold decrease in H2BK12cr in 3D4/21 cells after *T. gondii* infection, and the results of Western blot further confirmed the reduction of H2BK12cr levels (**Figures 3C, D** **)**. The above results supported that *T. gondii* infection negatively regulates histone Kcr in porcine alveolar macrophages.

### *T. gondii* Infection Inhibits H2BK12 Crotonylation *via* Epigenetic Modification

**Figure 4:**
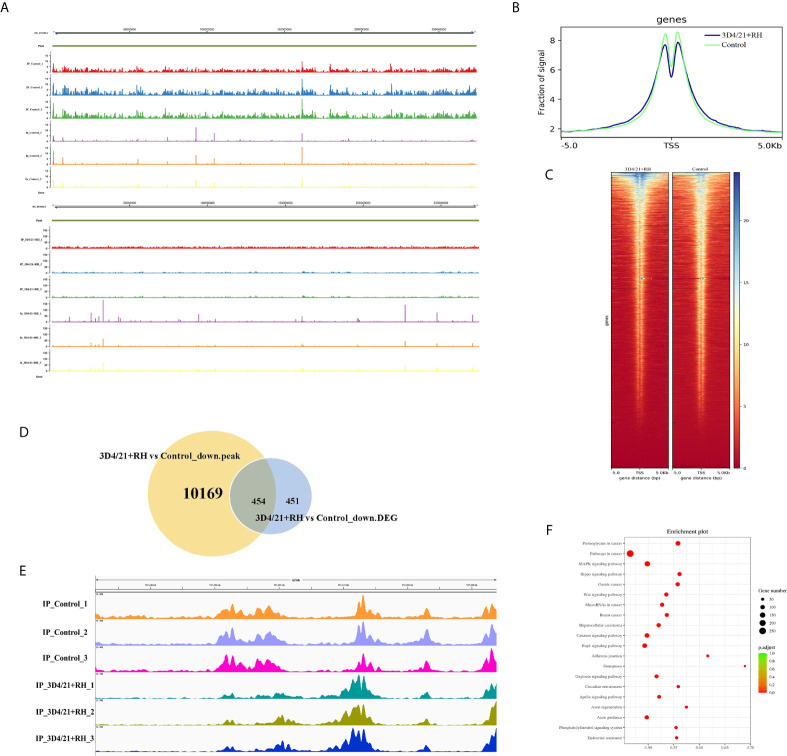
*T. gondii* leads to a decrease in H2BK12cr in porcine alveolar macrophages. **(A)** Browser view of a segment from chromosome NC_010443.5 showing a representative profile of the distribution of H2BK12cr peaks in 3D4/21+RH and control cells, respectively. **(B)** Average ChIP coverage levels of H2BK12cr in 3D4/21+RH and control cells at transcription sites. **(C)** ChIP-seq density heatmaps in 3D4/21+RH and control cells for H2BK12cr at TSS ± 5,000 bp. **(D)** Venn diagram showing the correlation analysis of downregulated peaks in ChIP-seq and downregulated DEGs in RNA-seq. **(E)** IGV enrichment tracks of H2BK12cr at USP4 in 3D4/21+RH and control cells, respectively. **(F)** Bubble diagram showing the KEGG pathways enriched by the top 20 genes with downregulated H2BK12cr.

To explore chromatin function of H2BK12 crotonylation, we performed anti-H2BK12cr chromatin immunoprecipitation-sequencing (ChIP-seq) assays of control and 3D4/21+RH cells. It could be observed from the results that H2BK12cr was well characterized in the genome with three replications (**Figure 4A**), and H2BK12cr levels peak in the vicinity of transcription start sites (TSS) (**Figures 4B, C** **)**. In order to explore the link between H2BK12cr and transcription, we combined the chip-seq with previously reported RNA-seq for conjoint analysis (36). We found that significant decreases in H2BK12cr levels of 454 genes synchronized with the reduction of transcription levels (more than 50% of the total significant downregulated genes), which further supported that *T. gondii* infection restrained H2BK12 crotonylation thereby inhibiting gene expression (**Figure 4D**). To further confirm the epigenetic regulation of H2BK12cr on *T. gondii* infection, we observed H2BK12cr modification levels were dramatically upregulated in the exon region of NF-*κ*B inhibitor zeta (NF-*κ*BIZ), which might imply the inhibition of NF-*κ*B (**Figure 4E**). Moreover, KEGG pathway analysis revealed that genes with downregulated levels of H2BK12cr were significantly enriched in transcriptional regulation- and proliferation-related pathways, such as MAPK signaling, Wnt signaling pathway, and Rap1 signaling pathway (**Figure 4F**).

### HDAC2 Primarily Regulates the Kcr Level in Porcine Alveolar Macrophages After *T. gondii* Infection

**Figure 5:**
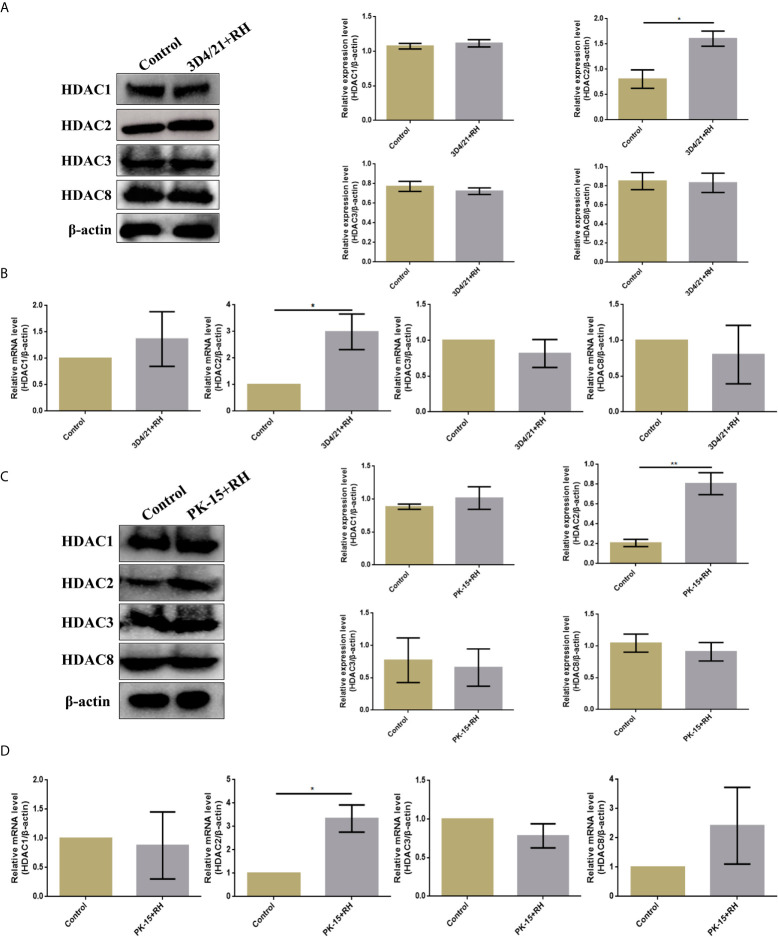
*T. gondii* leads to a decrease in HDAC2 in porcine alveolar macrophages and PK-15 cells. **(A)** Western blot analysis of the expression levels of class I HDACs in 3D4/21+RH and control cells. **(B)** RT-PCR analysis of the expression levels of class I HDACs in 3D4/21+RH and control cells. **(C)** Western blot analysis of the expression levels of class I HDACs in PK-15+RH and control cells. **(D)** RT-PCR analysis of the expression levels of class I HDACs in PK-15+RH and control cells. The data are presented as the mean ± SEM of three independent experiments. *p < 0.05, **p < 0.01, Student’s *t*-test.

**Figure 6:**
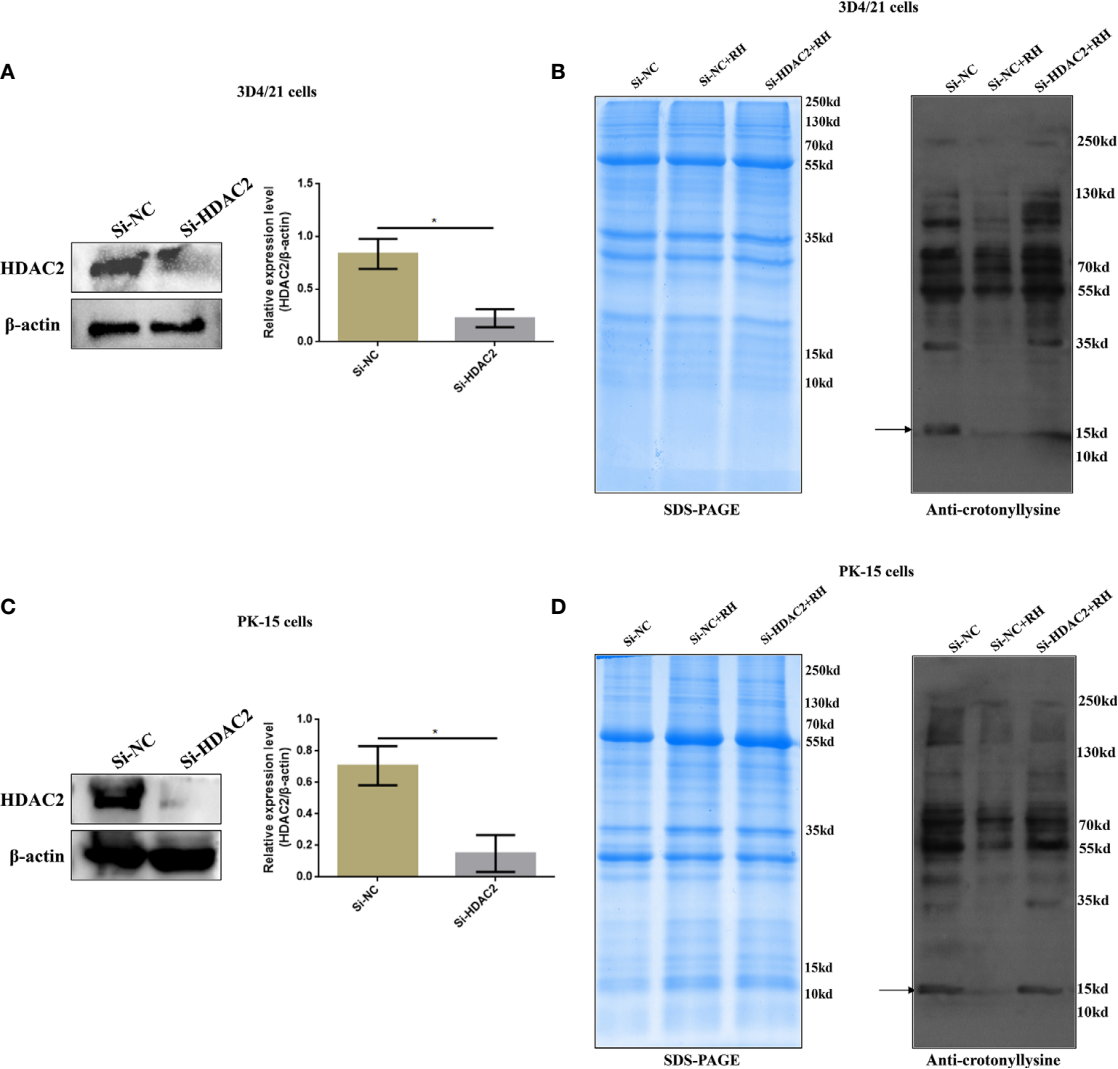
Effects of HDAC2 on crotonylation in porcine alveolar macrophages and PK-15 cells. **(A)** Western blot analysis of the expression levels of HDAC2 in Si-NC and Si-HDAC2 of 3D4/21 cells. **(B)** Western blot analysis showing the influence of HDAC2 knockdown on Kcr proteins levels in porcine alveolar macrophages. **(C)** Western blot analysis of the expression levels of HDAC2 in Si-NC and Si-HDAC2 of PK-15 cells. **(D)** Western blot analysis showing the influence of HDAC2 knockdown on Kcr proteins levels in PK-15 cells. *p < 0.05, Student’s *t*-test.

Given previous studies have indicated that class I histone deacetylases are the crucial regulators of histone crotonylation in multiple mammalian cells, which function as histone decrotonylases (24, 25). And HDACs are the primary epigenetic modulators involved in many human diseases. Hence, we measured the expression levels of class I histone deacetylases HDAC1, HDAC2, HDAC3, and HDAC8 in 3D4/21 cells and PK-15 cells after *T. gondii* infection, respectively. As shown in **Figure 5**, the *T. gondii* stimulation merely induced a significant increase of HDAC2 expression levels among class I histone deacetylases, which may imply the modulatory role of HDAC2. We next confirmed a regulation function of crotonylation of HDAC2 in porcine cells infected with *T. gondii*. Observably, Western blot analysis using pan-Kcr antibody revealed that the levels of Kcr in porcine cells with silence of HDAC2 after *T. gondii* infection were similar to that of uninfected cells (**Figure 6**), and we further observed that the modification levels of H2BK12cr can also be effectively recovered with silence of HDAC2, which indicated that HDAC2 was highly correlated with dynamic changes of Kcr induced by *T. gondii* stimulation (**Figure 3D**). Collectively, these data illustrated that the reduction in Kcr modification levels caused by *T. gondii* infection was indeed mediated *via* HDAC2, which might play a major role in histone decrotonylation in *T. gondii*-infected porcine alveolar macrophages.

### HDAC2-Regulated Histone Crotonylation Suppresses NF-κB Activation and Promotes Proliferation of Porcine Alveolar Macrophages Infected With *T. gondii*

**Figure 7:**
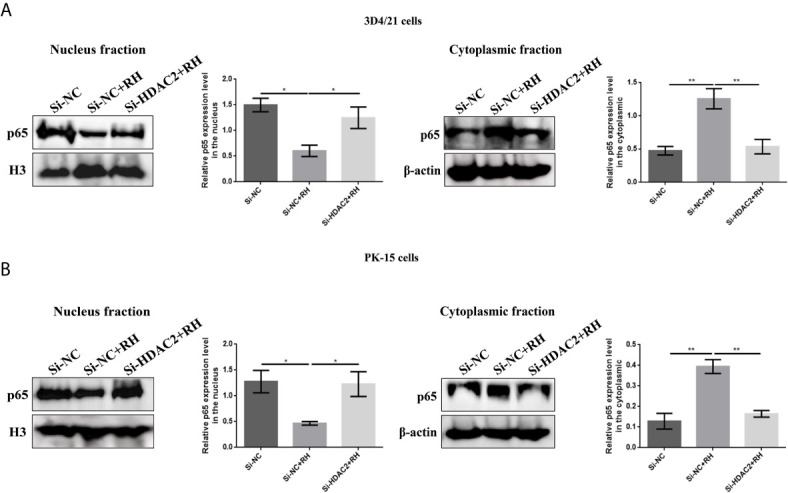
Effects of HDAC2-regulated histone crotonylation on NF-*κ*B activation. **(A)** The expression levels of NF-*κ*B p65 in 3D4/21 cells were measured by Western blotting. **(B)** The expression levels of NF-*κ*B p65 in PK-15 cells were measured by Western blotting. *p < 0.05, **p < 0.01, one-way ANOVA.

**Figure 8:**
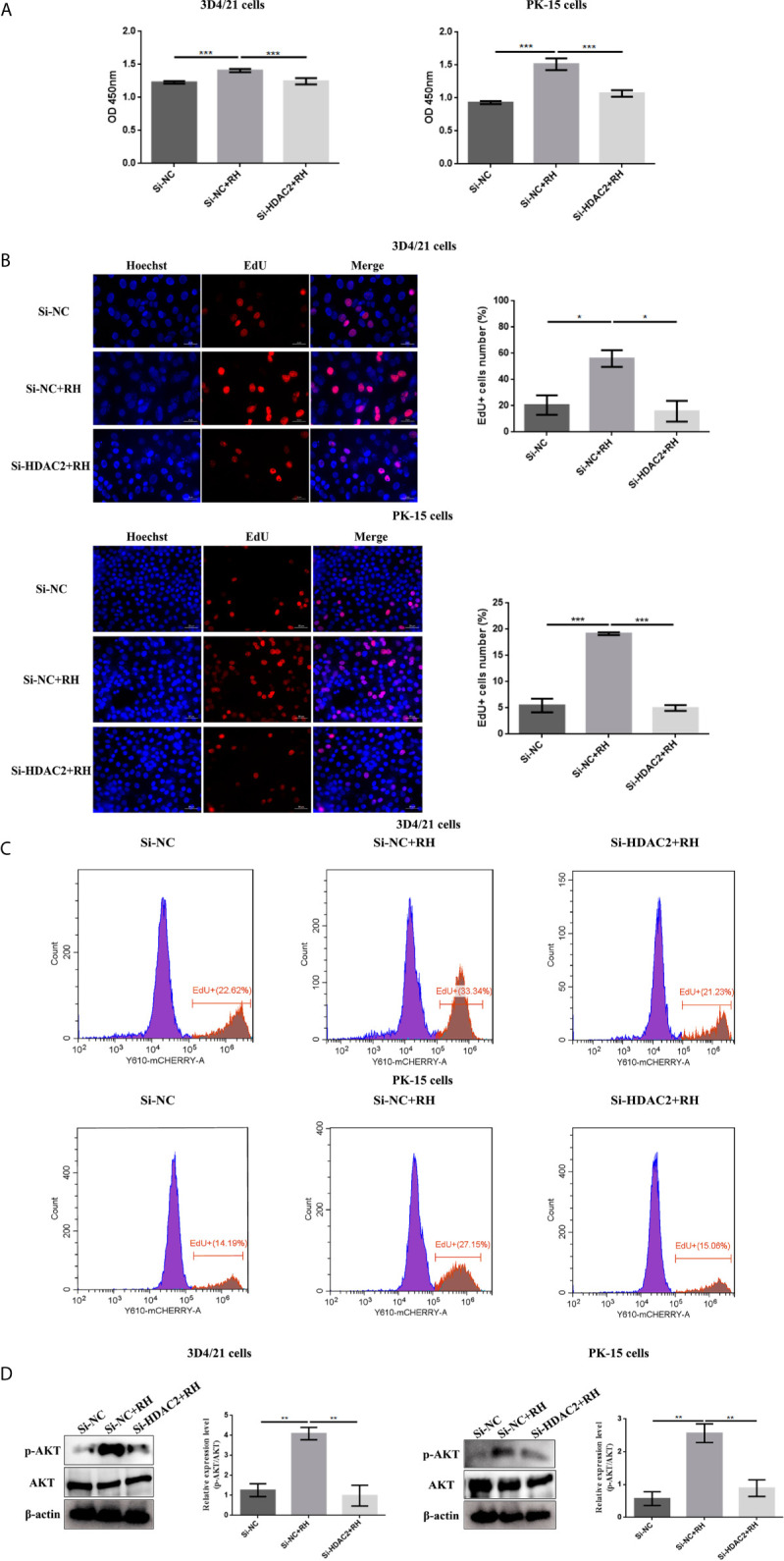
Effects of HDAC2-regulated histone crotonylation on cell proliferation after *T. gondii* infection. The effect of *T. gondii* infection on the macrophage proliferation was measured by CCK-8 **(A)** and EdU **(B, C)** assays. **(D)** The expression levels of Akt in 3D4/21 cells and PK-15 cells were measured by Western blotting. The data are presented as the mean ± SEM of three independent experiments. *p < 0.05, **p < 0.01, ***p < 0.001, one-way ANOVA.

NF-*κ*B is a family of dimeric transcription factors and plays an important role in immune responses modulated by parasite infection in macrophages. Given that H2BK12cr was significantly upregulated in the exon region of NF-*κ*BIZ, we next measured the activation of the NF-*κ*B pathway in cells after *Toxoplasma* infection. Our finding showed downregulated expression levels of NF-*κ*B p65 in nucleus extracts and upregulated expression levels of NF-*κ*B-p65 in cytoplasmic extracts of *T. gondii*-infected cells than those of control cells, while NF-*κ*B-p65 expression levels in HDAC2-silenced cells infected with *T. gondii* were consistent with those of uninfected cells (**Figure 7**). Overall, these data illustrated that histone crotonylation regulated by HDAC2 suppressed the activation of transcription factor NF-*κ*B. In order to further investigate whether histone Kcr modulated macrophage proliferation, we measured cell proliferation after parasite infection. As shown in **Figures 8A–C**, *T. gondii* infection enhanced cell proliferation, while silencing of HDAC2 expression neutralized the increases. Next, we analyzed which signaling pathway mediated macrophage proliferation caused by *T. gondii* infection. Previous studies have demonstrated that the activation of PI3K/Akt signaling pathway can promote cell proliferation (37, 38), and our results revealed that macrophage proliferation caused by *T. gondii* infection was indeed mediated *via* the activation of PI3K/Akt signaling pathway (**Figure 8D**).

## Discussion

Posttranslational modification (PTM) of protein can strictly regulate various cell biological processes by merely dictating protein function, localization, and stability. For the past few years, more than 400 PTMs related to cellular functions and diseases have been explored with the development of mass spectrometry technology (39). Previous research has substantiated that significant alterations of lysine acetylation occurred in cortical astrocytes after *T. gondii* infection (40). Lysine crotonylation is a new PTM discovered in 2011, which is completely different from the previously studied lysine acetylation in structure and function. Lysine crotonylation was first identified on histones, which was proven to play an essential role in regulating gene transcription (25, 41). Macrophages have been used as a model cell for the studies of the interaction between *T. gondii* and host cells (42). However, the biological functions of this newly identified PTM have not yet been reported in macrophages infected with *T. gondii*. Here we first conducted a comprehensive analysis of lysine crotonylation in porcine alveolar macrophages after *T. gondii* infection, which demonstrated extensive existence and significant difference of lysine crotonylation in porcine alveolar macrophages.

In the current study, we found that numerous proteins presented significant changes in Kcr levels, and especially histone Kcr levels were all dramatically downregulated after *T. gondii* infection. Therefore, we can infer that *Toxoplasma* infection inhibits histone Kcr modification. In addition, bioinformatics analysis was in accordance with our expectations, which revealed that downregulated Kcr proteins were basically enriched in gene transcription control pathways. In mammalian cells, HDACs comprise a family of 18 enzymes that are grouped in four typical categories (43). HDACs are first found to remove acetyl group from *ϵ*-N-acetyl lysine amino acids, and it is well recognized that HDACs are important in the epigenetic regulation of gene expression and the control of cell activities (44, 45). Unexpectedly, it has been reported that class I histone deacetylases are major executors of histone decrotonylation in mammalian cells, and selective HDACs are relevant to a broad transcription inhibition (25). Base on this, we speculated that parasite infection might affect the expression levels of class I histone deacetylases in porcine alveolar macrophages. It was found that merely HDAC2 expression levels in cells infected with *T. gondii* were observably increased. This observation, in conjunction with our results, indicates the importance of HDAC2 in modulating histone crotonylation in porcine alveolar macrophages, and the increase of HDAC2 could be the reason for the overall downregulation of histone crotonylation after *T. gondii* stimulation.

The PTM of histones plays a key role in epigenetic regulation and chromatin remodeling, thus broadly affecting gene expression (46). Crotonylation is first identified on histones, which not only in the N-terminal but also in various residues of the spherical domain is strongly modified (16). The positive charge of histone can reduce after the introduction of negatively charged crotonyl, which will lead to the looser binding of negatively charged DNA and is conducive to the binding of transcription factors. Many lines of evidence indicated that histone crotonylation has a role in regulating gene transcription, specifically marking the enhancers and transcription start site of genomic regions (16, 21). Widespread biological processes, including replication, transcription, and DNA damage repair are determined by histone modification, which represents important mechanism of epigenetic regulatory networks (41). Histone crotonylation has also been proved to play a catalytic role in epigenetic maintenance (47). Whereupon, we concluded that *T. gondii* infection might regulate epigenetics by altering histone Kcr modification. To data, it has been proven that regulatory enzymes of histone crotonylation are responsible for diversified lysine sites on histones, such as H3K18cr, H3K14cr, and H4K8cr (20, 24, 25). CDYL, as a Crotonyl-CoA hydratase, is demonstrated to negatively regulate histone Kcr, and CDYL depletion can lead to a significant increase in H2BK12cr level (41). In addition, the CoAP domain of CDYL has been shown to bind coenzyme A and recruit HDAC1 and HDAC2 (48). In apparent correspondence with our expectation, H2BK12cr expression level was notably decreased after *T. gondii* treatment, which might suggest in another aspect that H2BK12cr was a specific catalytic site for HDAC2. We observed a small but significant decrease in H2BK12cr in the vicinity of TSS in 3D4/21 cells infected with *T. gondii*, which controlled the expression of responsive genes. Conjoint analysis between Chip-seq and RNA-seq revealed that *T. gondii* infection could downregulate histone Kcr and thus restrain gene expression, which was consistent with a role for histone Kcr in positively correlating with gene expression (20).

Furthermore, we also observed that the enrichment of H2BK12cr on the exon region of NF-*κ*B inhibitor zeta was markedly increased, which may act as an inhibitor of NF-*κ*B (49). NF-κB is the vital component of innate and adaptive immunity, and the translocation of NF-κB to the nucleus can induce the expression of a wide range of genes in the immune response (50). Notably, the polymorphic rhoptry protein ROP18 in *T. gondii* type I strains can target the host p65 protein and cause its ubiquitin-dependent degradation, thereby preventing the nuclear translocation of p65 and inhibiting the NF-κB pathway (15). Furthermore, Toxoplasma can regulate the NF-κB pathway according to the parasite genotype and host cell lineage, and EZH2 can facilitate the epigenetic silencing of NF-κB thereby construing to the parasite persistence in mice (51). More evidence shows that there is a regulatory relationship between HDAC2 and NF-κB. Previous research suggests that HDAC2 can function to negatively regulate NF-κB transcriptional activity and that HDAC2 inhibitor leads to increased expression of NF-κB-dependent reporter genes with indirect interaction after TSA stimulation (52). HDAC2 has also been confirmed to specifically recruit NF-κB at the target promoter and subsequently affects acetylation, which may play an important role in regulating iNOS and other NF-κB-dependent genes involved in inflammation (53). Additionally, HDAC2 knockdown enhances the activity of NF-κBp65, thereby increasing IL-8 and TNF-α levels in murine emphysema model (54). USP4, a target gene of HDAC2, can downregulate TNF-α-induced NF-κB activation (55). Therefore, we inferred that *T. gondii* RH strain could inhibit NF-κB activation, which was regulated by HDAC2 that could mediate histone crotonylation.

KEGG pathways suggested that genes with downregulated H2BK12cr were enriched in diversified pathways related to cell proliferation. Indeed, we demonstrated that *T. gondii* infection could promote macrophage proliferation. However, it was unexpected that *T. gondii* stimulation promoted proliferation by reducing histone crotonylation. PI3K/Akt signaling pathway has been demonstrated to be involved in a variety of biological processes, which are critical to mediate various cell functions, including metabolism, apoptosis, cell growth, and survival (56, 57). An increasing number of evidence have shown that PI3K/Akt signaling pathway plays an important role in host cells infected with *T. gondii*, and the activation of host PI3K/Akt pathway can suppress the apoptosis of host cells to achieve parasite survival (58, 59). In previous research, Akt has been shown to be a downstream protein of HDAC2 that is positively regulated by HDAC2 in isoproterenol-induced cardiac hypertrophy (60). Besides, neuropathic pain stimulation can increase the expression level of HDAC2 and activate the PI3K/Akt/GSK-3*β* signaling pathway, and bone marrow macrophages (BMMs) show increased HDAC2 expression during osteoclastogenesis, thereby activating Akt and inhibiting FOXO1 (61, 62). Together the above studies fully support that Akt as a downstream target of HDAC2 is positively regulated by HDAC2. Next, we explored the potential mechanism of parasite-induced macrophage proliferation. Our data indicated that *T. gondii* infection-mediated histone Kcr was required for HDAC2-mediated activation of PI3K/Akt pathway, which might be the major reason for macrophage proliferation.

**Figure 9:**
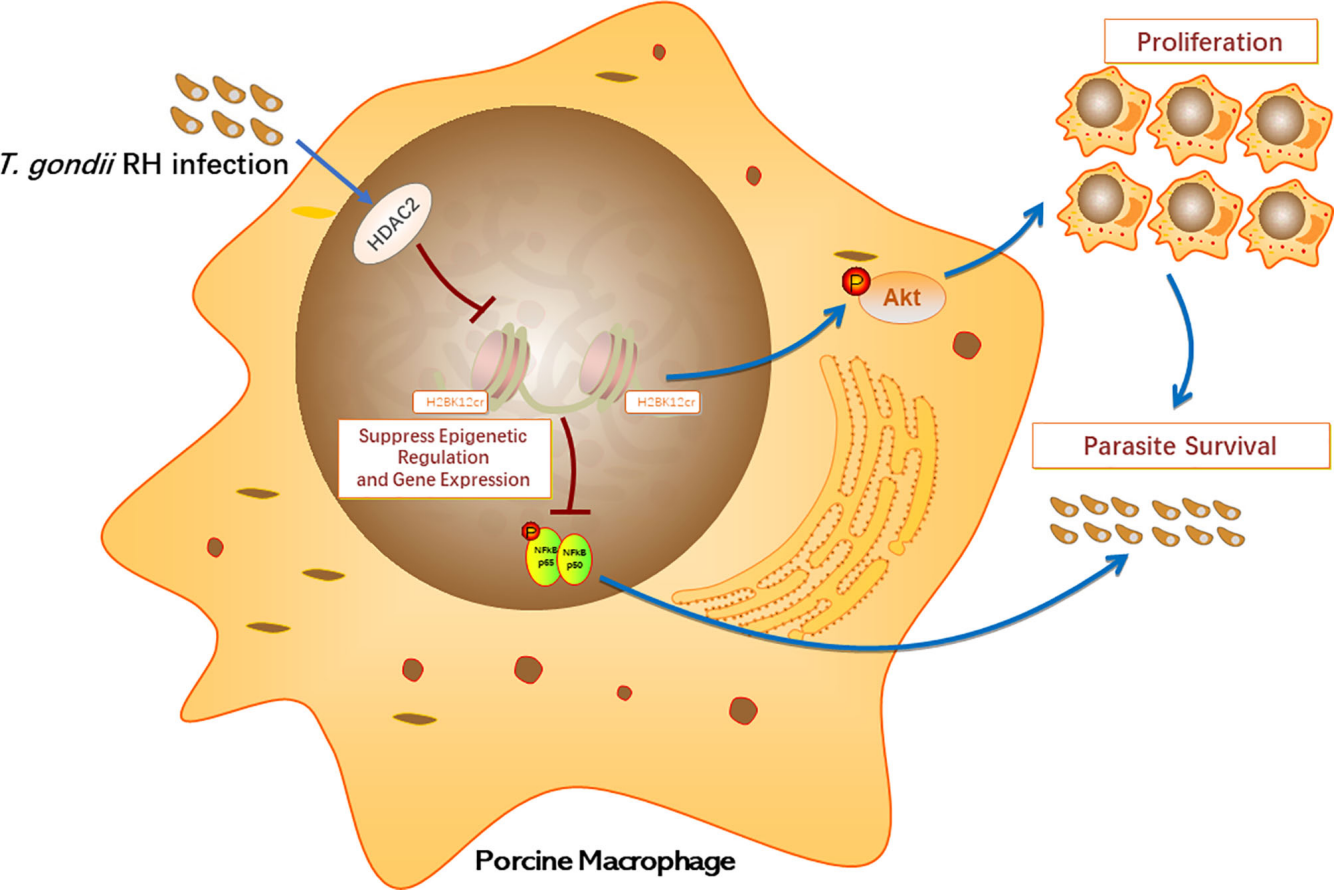
Schematic diagram of biological functions of histone crotonylation in the immune response of porcine alveolar macrophages against *T. gondii* infection.

In conclusion, our research provides a comprehensive understanding of the biological processes of histone crotonylation in porcine alveolar macrophages after *T. gondii*. To our knowledge, our study firstly revealed the existence of crotonylation in porcine alveolar macrophages. HDAC2 is the major histone decrotonylase in porcine alveolar macrophages infected with *T. gondii*. The expression levels of HDAC2 significantly increase due to *T. gondii* infection, thereby inhibiting histone crotonylation modification. In addition, parasite infection can control epigenetics to reduce the levels of transcription factor NF-κB by inhibiting H2BK12 crotonylation, but it in turn activates the PI3K/Akt pathway to promote host cell proliferation, contributing to parasite survival in host cells (**Figure 9**). Unveiling of the functions of histone Kcr can provide a certain research basis for the mechanism research on the immune response of host against *T. gondii*, which may also provide novel insights into the evasion mechanisms of parasite immune evasion.

## Data Availability Statement

The datasets presented in this study can be found in online repositories. The names of the repository/repositories and accession number(s) can be found in the article/**Supplementary Material**.

## Author Contributions

JZ and RF conceived and designed the study, JY, ZH, CC and SL performed the experiments, JY, ZH and JQ analyzed the data, and JY drafted the manuscript. All authors contributed to the article and approved the submitted version.

## Funding

This work was supported by the Independent Technology Innovation Fund Project of Huazhong Agricultural University (2662020DKPY015) and the European Union’s Horizon 2020 Research and Innovation program under grant agreement 773830: One Health European Joint Program.

## Conflict of Interest

The authors declare that the research was conducted in the absence of any commercial or financial relationships that could be construed as a potential conflict of interest.

## Supplementary Material

Supplementary Figure 1 Quantitative crotonylation analyses of porcine alveolar macrophages. **(A)** Overview of crotonylation modification identification. **(B)** Mass error distribution of all crotonylated peptides. **(C)** Distribution of lysine crotonylation peptides based on their length.

Supplementary Table 1 Complete list of quantitative Kcr sites identified in 3D4/21+RH and control cells.

Supplementary Table 2 Differentially expressed Kcr proteins identified in 3D4/21+RH and control cells.

## References

[1] Foe IT Child MA Majmudar JD Krishnamurthy S Bogyo M. Global Analysis of Palmitoylated Proteins in Toxoplasma Gondii. Cell Host Microbe (2015) 18(4):501–11. 10.1016/j.chom.2015.09.006 PMC4694575 26468752

[2] Lourido S. Toxoplasma Gondii. Trends Parasitol (2019) 35(11):944–5. 10.1016/j.pt.2019.07.001 31345768

[3] Sasai M Pradipta A Yamamoto M. Host Immune Responses to Toxoplasma Gondii. Int Immunol. (2018) 30(3):113–9. 10.1093/intimm/dxy004 29408976

[4] Hunter CA Sibley LD. Modulation of Innate Immunity by Toxoplasma Gondii Virulence Effectors. Nat Rev Microbiol (2012) 10(11):766–78. 10.1038/nrmicro2858 PMC3689224 23070557

[5] Nissapatorn V. Toxoplasma Gondii and HIV: A Never-Ending Story. Lancet HIV (2017) 4(4):e146–e7. 10.1016/S2352-3018(17)30003-6 28159547

[6] Han K Shin D-W Lee T-Y Lee Y-H. Seroprevalence of Toxoplasma Gondii Infection and Risk Factors Associated With Seropositivity of Pregnant Women in Korea. J Parasitol 94(4):963–5. 10.1645/GE-1435.1 18576787

[7] Casado I Domínguez Á Toledo D Chamorro J Astray J Egurrola M. Repeated Influenza Vaccination for Preventing Severe and Fatal Influenza Infection in Older Adults: A Multicentre Case–Control Study. Cmaj (2018) 190(1):E3–E12. 10.1503/cmaj.170910 29311098 PMC5760253

[8] Gisbert Algaba I Verhaegen B Jennes M Rahman M Coucke W Cox E. Pork as a Source of Transmission of Toxoplasma Gondii to Humans: A Parasite Burden Study in Pig Tissues After Infection With Different Strains of Toxoplasma Gondii as a Function of Time and Different Parasite Stages. Int J Parasitol. (2018) 48(7):555–60. 10.1016/j.ijpara.2017.12.009 29625125

[9] Julia N Kathrin ES Johannes W Henrike S-BK Horst S Christian S. Experimental Porcine *Toxoplasma Gondii* Infection as a Representative Model for Human Toxoplasmosis. Mediators Inflamm (2017) 2017:1–10. 10.1155/2017/3260289 PMC5572617 28883687

[10] Spetzler VN Goldaracena N Knaak JM Louis KS Selzner N Selzner M. Technique of Porcine Liver Procurement and Orthotopic Transplantation Using an Active Porto-Caval Shunt. J Visualized Exp (2015) 99:e52055. 10.3791/52055 PMC4542501 25992583

[11] Thorne KJI Blackwell JM. Cell-Mediated Killing of Protozoa. AdvParasitol (1983) 22:43–151. 10.1016/S0065-308X(08)60461-3 6364738

[12] Pifer R Yarovinsky F. Innate Responses to Toxoplasma Gondii in Mice and Humans. Trends Parasitol (2011) 27(9):388–93. 10.1016/j.pt.2011.03.009 PMC3159709 21550851

[13] Sibley LD Weidner E Krahenbuhl JL. Phagosome Acidification Blocked by Intracellular Toxoplasma Gondii. Nature (1985) 315(6018):416–9. 10.1038/315416a0 2860567

[14] Butcher BA Kim L Johnson PF Denkers EY. Toxoplasma Gondii Tachyzoites Inhibit Proinflammatory Cytokine Induction in Infected Macrophages by Preventing Nuclear Translocation of the Transcription Factor NF-κb. J Immunol (2001) 167(4):2193–201. 10.4049/jimmunol.167.4.2193 11490005

[15] Du J An R Chen L Shen Y Chen Y Cheng L. Toxoplasma Gondii Virulence Factor ROP18 Inhibits the Host NF-κb Pathway by Promoting P65 Degradation. J Biol Chem (2014) 289(18):12578–92. 10.1074/jbc.M113.544718 PMC4007449 24648522

[16] Tan M Luo H Lee S Jin F Yang JS Montellier E. Identification of 67 Histone Marks and Histone Lysine Crotonylation as a New Type of Histone Modification. Cell (2011) 146(6):1016–28. 10.1016/j.cell.2011.08.008 PMC3176443 21925322

[17] Deribe YL Pawson T Dikic I. Post-Translational Modifications in Signal Integration. Nat Struct Mol Biol (2010) 17(6):666–72. 10.1038/nsmb.1842 20495563

[18] Xu W Wan J Zhan J Li X He H Shi Z. Global Profiling of Crotonylation on non-Histone Proteins. Cell Res. (2017) 27(7):946–9. 10.1038/cr.2017.60 10.1038/cr.2017.60 PMC5518986 28429772

[19] Li Y Sabari BR Panchenko T Wen H Zhao D Guan H. Molecular Coupling of Histone Crotonylation and Active Transcription by AF9 YEATS Domain. Mol Cell (2016) 62(2):181–93. 10.1016/j.molcel.2016.03.028 PMC4841940 27105114

[20] Liu S Xue C Fang Y Chen G Peng X Zhou Y. Global Involvement of Lysine Crotonylation in Protein Modification and Transcription Regulation in Rice. Mol Cell Proteomics (2018) 17(10):1922–36. 10.1074/mcp.RA118.000640 PMC6166680 30021883

[21] Sabari BR Tang Z Huang H Yong-Gonzalez V Molina H Kong HE. Intracellular Crotonyl-CoA Stimulates Transcription Through P300-Catalyzed Histone Crotonylation. Mol Cell (2015) 58(2):203–15. 10.1016/j.molcel.2015.02.029 PMC4501262 25818647

[22] Liu X Wei W Liu Y Yang X Wu J. MOF as an Evolutionarily Conserved Histone Crotonyltransferase and Transcriptional Activation by Histone Acetyltransferase-Deficient and Crotonyltransferase-Competent CBP/p300. Cell Discov (2017) 3:17016. 10.1038/celldisc.2017.16 28580166 PMC5441097

[23] Bao X Wang Y Li X Li X-M Liu Z Yang T. Identification of ‘Erasers’ for Lysine Crotonylated Histone Marks Using a Chemical Proteomics Approach. Elife (2014) 3:e02999. 10.7554/eLife.02999 PMC4358366 25369635

[24] Kelly RDW Chandru A Watson PJ Song Y Blades M Robertson NS. Histone Deacetylase (HDAC) 1 and 2 Complexes Regulate Both Histone Acetylation and Crotonylation *In Vivo* . Sci Rep (2018) 8(1):14690–. 10.1038/s41598-018-32927-9 PMC6168483 30279482

[25] Wei W Liu X Chen J Gao S Lu L Zhang H. Class I Histone Deacetylases Are Major Histone Decrotonylases: Evidence for Critical and Broad Function of Histone Crotonylation in Transcription. Cell Res (2017) 27(7):898–915. 10.1038/cr.2017.68 28497810 PMC5518989

[26] Lührs H Gerke T Müller JG Melcher R Schauber J Boxberge F. Butyrate Inhibits NF-kappaB Activation in Lamina Propria Macrophages of Patients With Ulcerative Colitis. Scand J Gastroenterol (2002) 37(4):458–66. 10.1080/003655202317316105 11989838

[27] Urnov FD Wolffe AP. Chromatin Remodeling and Transcriptional Activation: The Cast (in Order of Appearance). Oncogene (2001) 20(24):2991–3006. 10.1038/sj.onc.1204323 11420714

[28] Fellows R Denizot J Stellato C Cuomo A Jain P Stoyanova E. Microbiota Derived Short Chain Fatty Acids Promote Histone Crotonylation in the Colon Through Histone Deacetylases. Nat Commun (2018) 9(1):105. 10.1038/s41467-017-02651-5 PMC5760624 29317660

[29] Yin D Jiang N Zhang Y Wang D Sang X Feng Y. Global Lysine Crotonylation and 2-Hydroxyisobutyrylation in Phenotypically Different Toxoplasma Gondii Parasites. Mol Cell Proteomics (2019) 18(11):2207–24. 10.1074/mcp.RA119.001611 PMC6823851 31488510

[30] Shechter D Dormann HL Allis CD Hake SB. Extraction, Purification and Analysis of Histones. Nat Protoc (2007) 2(6):1445–57. 10.1038/nprot.2007.202 17545981

[31] Robinson JT Thorvaldsdóttir H Winckler W Guttman M Lander ES Getz G. Integrative Genomics Viewer. Nat Biotechnol (2011) 29(1):24–6. 10.1038/nbt.1754 PMC3346182 21221095

[32] Svinkina T Gu H Silva JC Mertins P Qiao J Fereshetian S. Deep, Quantitative Coverage of the Lysine Acetylome Using Novel Anti-Acetyl-Lysine Antibodies and an Optimized Proteomic Workflow. Mol Cell Proteomics (2015) 14(9):2429–40. 10.1074/mcp.O114.047555 PMC4563726 25953088

[33] Park J Chen Y Tishkoff DX Peng C Tan M Dai L. SIRT5-Mediated Lysine Desuccinylation Impacts Diverse Metabolic Pathways. Mol Cell (2013) 50(6):919–30. 10.1016/j.molcel.2013.06.001 PMC3769971 23806337

[34] Berdasco M Esteller M. Aberrant Epigenetic Landscape in Cancer: How Cellular Identity Goes Awry. Dev Cell (2010) 19(5):0–711. 10.1016/j.devcel.2010.10.005 21074720

[35] Yamauchi Y Cooper P Shimizu E Kobayashi Y Smith A Duncan H. Histone Acetylation as a Regenerative Target in the Dentine-Pulp Complex. Front Genet (2020) 11:1–8. 10.3389/fgene.2020.00001 32117431 PMC7016267

[36] Song Y Song L Wan X Shen B Fang R Hu M. A Comparison of Transcriptional Diversity of Swine Macrophages Infected With TgHB1 Strain of Toxoplasma Gondii Isolated in China. Front Cell Infect Microbiol (2020) 10:526876. 10.3389/fcimb.2020.526876 33102248 PMC7546811

[37] Gong C Ai J Fan Y Gao J Liu W Feng Q. NCAPG Promotes The Proliferation Of Hepatocellular Carcinoma Through PI3K/AKT Signaling. OncoTargets Ther (2019) 12:8537–52. 10.2147/ott.s217916 PMC6801502 31802891

[38] Woeller CF Roztocil E Hammond C Feldon SE. TSHR Signaling Stimulates Proliferation Through PI3K/Akt and Induction of miR-146a and miR-155 in Thyroid Eye Disease Orbital Fibroblasts. Invest Ophthalmol Visual Sci (2019) 60(13):4336–45. 10.1167/iovs.19-27865 PMC6798326 31622470

[39] Aebersold R Mann M. Mass-Spectrometric Exploration of Proteome Structure and Function. Nature (2016) 537(7620):347–55. 10.1038/nature19949 27629641

[40] Bouchut A Chawla AR Jeffers V Hudmon A Sullivan WJ Jr. Proteome-Wide Lysine Acetylation in Cortical Astrocytes and Alterations That Occur During Infection With Brain Parasite Toxoplasma Gondii. PloS One (2015) 10(3):e0117966. 10.1371/journal.pone.0117966 25786129 PMC4364782

[41] Liu S Yu H Liu Y Liu X Zhang Y Bu C Chromodomain Protein CDYL Acts as a Crotonyl-CoA Hydratase to Regulate Histone Crotonylation and Spermatogenesis. Mol Cell (2017) 67(5):853–66. 10.1016/j.molcel.2017.07.011 28803779

[42] Delgado Betancourt E Hamid B Fabian BT Klotz C Hartmann S Seeber F. From Entry to Early Dissemination-Toxoplasma Gondii’s Initial Encounter With Its Host. Front Cell Infect Microbiol (2019) 9:46. 10.3389/fcimb.2019.00046 30891433 PMC6411707

[43] Hull EE Montgomery MR Leyva KJ. HDAC Inhibitors as Epigenetic Regulators of the Immune System: Impacts on Cancer Therapy and Inflammatory Diseases. BioMed Res Int (2016) 2016:8797206. 10.1155/2016/8797206 27556043 PMC4983322

[44] Longworth MS Laimins LA. Histone Deacetylase 3 Localizes to the Plasma Membrane and Is a Substrate of Src. Oncogene (2006) 25(32):4495–500. 10.1038/sj.onc.1209473 16532030

[45] Gräff J Tsai LH. The Potential of HDAC Inhibitors as Cognitive Enhancers. Annu Rev Pharmacol Toxicol (2013) 53:311–30. 10.1146/annurev-pharmtox-011112-140216 23294310

[46] Brien GL Valerio DG Armstrong SA. Exploiting the Epigenome to Control Cancer-Promoting Gene-Expression Programs. Cancer Cell (2016) 29(4):464–76. 10.1016/j.ccell.2016.03.007 PMC4889129 27070701

[47] Xiong X Panchenko T Yang S Zhao S Yan P Zhang W. Selective Recognition of Histone Crotonylation by Double PHD Fingers of MOZ and DPF2. Nat Chem Biol (2016) 12: (12):1111–8. 10.1038/nchembio.2218 PMC5253430 27775714

[48] Caron C Pivot-Pajot C van Grunsven LA Col E Lestrat C Rousseaux S. Cdyl: A New Transcriptional Co-Repressor. EMBO Rep (2003) 4(9):877–82. 10.1038/sj.embor.embor917 PMC1326355 12947414

[49] Yamazaki S Muta T Takeshige K. A Novel IkappaB Protein, IkappaB-Zeta, Induced by Proinflammatory Stimuli, Negatively Regulates Nuclear factor-kappaB in the Nuclei. J Biol Chem (2001) 276(29):27657–62. 10.1074/jbc.M103426200 11356851

[50] Gilmore TD Wolenski FS. NF-κb: Where did it Come From and Why? Immunol Rev (2012) 246(1):14–35. 10.1111/j.1600-065X.2012.01096.x 22435545

[51] Braun L Brenier-Pinchart M-P Hammoudi P-M Cannella D Kieffer-Jaquinod S Vollaire J. The Toxoplasma Effector TEEGR Promotes Parasite Persistence by Modulating NF-κb Signalling *via* EZH2. Nat Microbiol (2019) 4(7):1208–20. 10.1038/s41564-019-0431-8 PMC6591128 31036909

[52] Ashburner BP Westerheide SD Baldwin AS Jr. The P65 (RelA) Subunit of NF-kappaB Interacts With the Histone Deacetylase (HDAC) Corepressors HDAC1 and HDAC2 to Negatively Regulate Gene Expression. Mol Cell Biol (2001) 21(20):7065–77. 10.1128/mcb.21.20.7065-7077.2001 PMC99882 11564889

[53] Yu Z Zhang W Kone BC. Histone Deacetylases Augment Cytokine Induction of the iNOS Gene. J Am Soc Nephrol: JASN (2002) 13(8):2009–17. 10.1097/01.asn.0000024253.59665.f1 12138131

[54] Bin Y Xiao Y Huang D Ma Z Liang Y Bai J. Theophylline Inhibits Cigarette Smoke-Induced Inflammation in Skeletal Muscle by Upregulating HDAC2 Expression and Decreasing NF-κb Activation. Am J Physiol Lung Cell Mol Physiol (2019) 316(1):L197–205. 10.1152/ajplung.00005.2018 30358442

[55] Li Z Hao Q Luo J Xiong J Zhang S Wang T. USP4 Inhibits P53 and NF-κb Through Deubiquitinating and Stabilizing HDAC2. Oncogene (2016) 35(22):2902–12. 10.1038/onc.2015.349 PMC4895393 26411366

[56] Ehrhardt C Ludwig S. A New Player in a Deadly Game: Influenza Viruses and the PI3K/Akt Signalling Pathway. Cell Microbiol (2009) 11(6):863–71. 10.1111/j.1462-5822.2009.01309.x PMC7162392 19290913

[57] Yu JSL Cui W. Proliferation, Survival and Metabolism: The Role of PI3K/AKT/mTOR Signalling in Pluripotency and Cell Fate Determination. Development (2016) 143(17):3050. 10.1242/dev.137075 27578176

[58] Zhou W Quan J-H Lee Y-H Shin D-W Cha G-H. Toxoplasma Gondii Proliferation Require Down-Regulation of Host Nox4 Expression *via* Activation of PI3 Kinase/Akt Signaling Pathway. PloS One (2013) 8(6):e66306–e. 10.1371/journal.pone.0066306 PMC3688893 23824914

[59] Chen J Hu L Wang J Cao Y Zhu D Chen L. Toxoplasma Gondii Excreted-Secreted Antigens Suppress Foxp3 *via* PI3K-AKT-mTOR Signaling Pathway. J Cell Biochem (2019) 120(9):16044–51. 10.1002/jcb.28884 31074049

[60] Shang L Pin L Zhu S Zhong X Zhang Y Shun M. Plantamajoside Attenuates Isoproterenol-Induced Cardiac Hypertrophy Associated With the HDAC2 and AKT/GSK-3β Signaling Pathway. Chem-Biol Interact (2019) 307:21–8. 10.1016/j.cbi.2019.04.024 31009642

[61] Yuan L Liu C Wan Y Yan H Li T. Effect of HDAC2/Inpp5f on Neuropathic Pain and Cognitive Function Through Regulating PI3K/Akt/GSK-3β Signal Pathway in Rats With Neuropathic Pain. Exp Ther Med (2019) 18(1):678–84. 10.3892/etm.2019.7622 PMC6580097 31281447

[62] Dou C Li N Ding N Liu C Yang X Kang F. HDAC2 Regulates FoxO1 During RANKL-Induced Osteoclastogenesis. Am J Physiol Cell Physiol (2016) 310(10):C780–7. 10.1152/ajpcell.00351.2015 26962001

