# Supplementary figures and images for: Toxoplasma gondii Infection Inhibits Histone Crotonylation to Regulate Immune Response of Porcine Alveolar Macrophages
Source: Front Immunol. 2021 Jul 8;12:696061. doi: 10.3389/fimmu.2021.696061 (PMC8312545; doi:10.3389/fimmu.2021.696061)

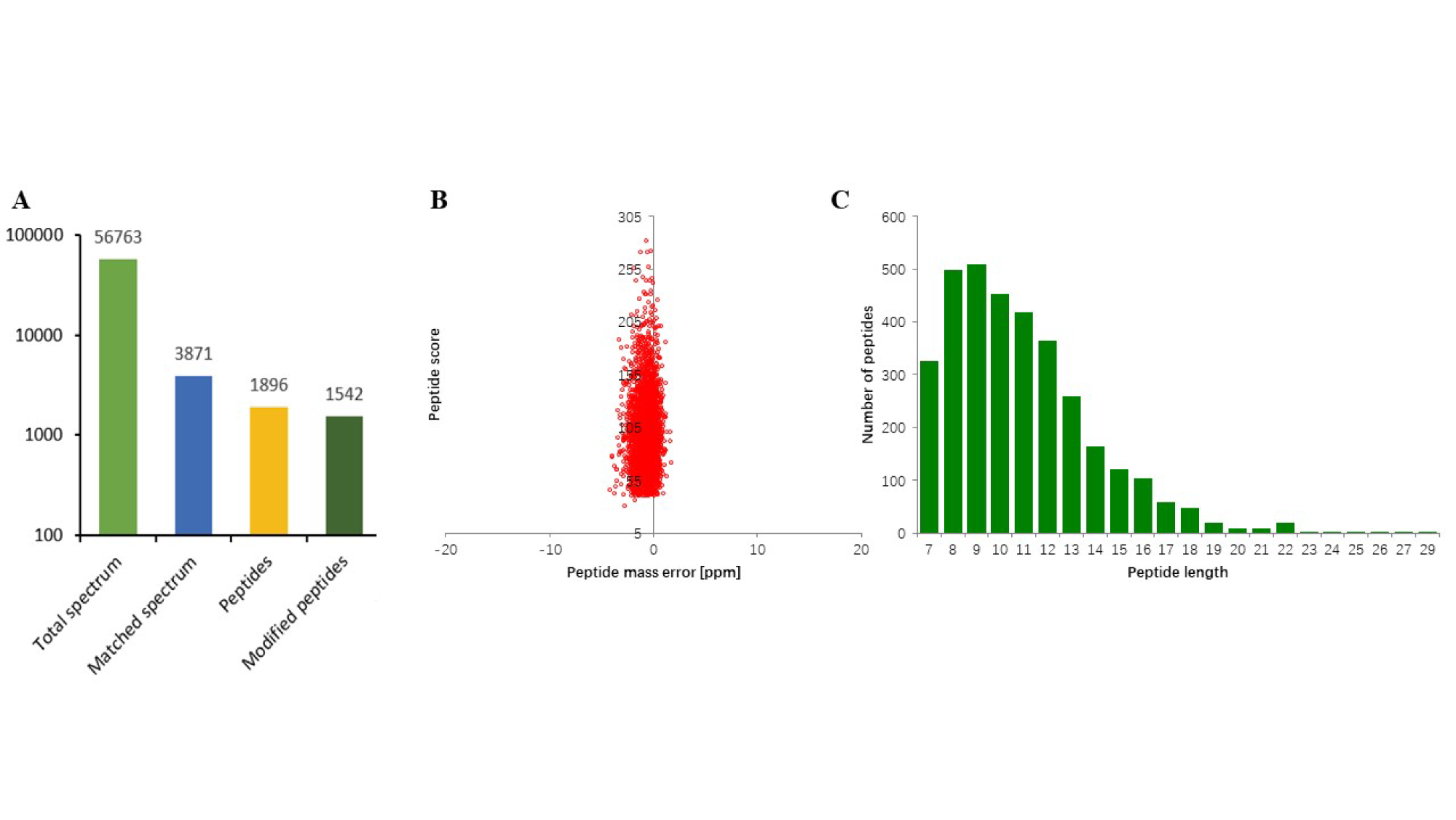

Supplement: Supplementary Figure 1 — Quantitative crotonylation analyses of porcine alveolar macrophages. (A) Overview of crotonylation modification identification. (B) Mass error distribution of all crotonylated peptides. (C) Distribution of lysine crotonylation peptides based on their length. [file Image_1.tif]
